# Bidah Pomegranate Landrace: Chemical Composition, Antioxidant, Antibacterial, and Anticancer Activity

**DOI:** 10.3390/life15030489

**Published:** 2025-03-18

**Authors:** Abdalrhaman M. Salih, Nada M. Alattas, Qasi D. Alsubaie, Saheed O. Anifowose

**Affiliations:** 1 National Research and Development Center for Sustainable Agriculture (Estidamah), Riyadh Techno Valley, Riyadh 12373, Saudi Arabia; 441200966@pnu.edu.sa (N.M.A.); sabieyq@estidamah.gov.sa (Q.D.A.); 2 Botany and Microbiology Department, College of Science, King Saud University, P.O. Box 2455, Riyadh 11451, Saudi Arabia; 3 Zoology Department, College of Science, King Saud University, P.O. Box 2455, Riyadh 11451, Saudi Arabia; 444105896@student.ksu.edu.sa

**Keywords:** Bidah pomegranate cultivar, shoot, callus, antioxidant capacity, antibacterial, apoptosis

## Abstract

Pomegranate (*Punica granatum* L.) belongs to the Punicaceae family and is native to Central Asia; yet, it has a wide geographical distribution globally, reflecting its adaptation to different climatic conditions. Pomegranate is among the oldest and most significant cultivated crops, thriving extensively in tropical and subtropical climates. Besides its nutritional uses, pomegranate has been employed in traditional medicine for treating various diseases, including cancer prevention, antimicrobial activity, male infertility, ulcers, and diarrhea. The Bidah pomegranate cultivar is known for its unique sweet taste and high productivity yield. However, there is limited knowledge about its nutritional composition and medicinal value. Therefore, this study aimed to evaluate the functional potential of Bidah pomegranate in terms of its phytochemicals, antioxidant capacity, antibacterial, and anticancer activity. Different analytical techniques were used to investigate the chemical composition and antioxidant properties of Bidah pomegranate. Moreover, the biological activity of shoot and callus of Bidah pomegranate cultivar was assessed against *Escherichia coli* (*E. coli*), *Staphylococcus aureus* (*S. aureus*), and the colorectal cancer cell line (CaCo-2). Gas chromatography/mass spectrometry (GC/MS) analysis of the shoot and callus extracts revealed about 17 and 18 phytochemical compounds, respectively. Phenolic quantification showed that pomegranate materials contained high amounts of phenolic content. Additionally, Bidah pomegranate cultivar possesses high antioxidant activity with a low half-maximal inhibitory concentration (IC50) value. Furthermore, the pomegranate extract showed promising results with human pathogenic bacteria (*E. coli* and *S. aureus*), especially against *S. aureus* and the colorectal cancer cell line (CaCo-2). The findings of this study support the traditional use of pomegranate in folk medicine and highlight its potential for further exploration as a source of therapeutic agents.

## 1. Introduction

Pomegranate (*Punica granatum* L.) is a large shrub or small tree that belongs to the Punicaceae family. It is an endemic plant of Iran and is also found in northern India, China, the USA, and throughout the Mediterranean region [1,2]. Pomegranate is one of the medicinal plants that has been used for treating different types of diseases in folk medicine, such as in anti-inflammatory contexts, diarrhea, ulcers, and male infertility [3,4]. Moreover, pomegranate possesses a wide range of pharmacological activities such as anti-diabetic, antitumor, anti-inflammatory, anti-malaria, anti-fibrotic, antifungal, antibacterial, and other effects [5,6]. In addition, various parts of the pomegranate plant have been tested for their potential medicinal advantages in treating conditions such as cancer, cardiovascular problems, gum infections, and diabetes [7,8]. Pomegranates are rich in bioactive compounds such as flavonoids, flavones, flavonol, anthocyanidins, ellagic acid, anthocyanins, ellagitannins, and punicic acid. Laboratory data suggest that these compounds offer various potential health benefits [9]. Additionally, pomegranate exhibits broad-spectrum antibacterial activity against both Gram-positive and Gram-negative bacteria. The presence of ellagitannins and other polyphenolic compounds in the pomegranate extracts contributes to its antimicrobial properties [10,11]. This makes pomegranate a promising natural alternative for combating bacterial infections and reducing the reliance on synthetic antibiotics. Moreover, pomegranate extracts have been shown to inhibit the growth of cancer cells and induce apoptosis through multiple cellular and molecular mechanisms. The presence of compounds such as punicalagin, ellagic acid, and anthocyanins plays a crucial role as an anticancer agent [12,13]. Furthermore, the consumption of pomegranate could be used to improve gut microbiota and therefore prevent obesity and diabetes [4,6].

Bidah pomegranate cultivar is a valuable fruit crop grown in the Al-Baha region of Saudi Arabia, particularly in the Bidah area. This cultivar has a unique sweet taste and high productivity yield. Based on the DNA barcode, Bidah pomegranate is a distinct cultivar genetically and may adapt to the location [14]. On the other hand, medicinal plants have been used in the treatment of many diseases. For example, the plant secondary metabolites have a significant inhibitory effect on the growth of pathogenic bacteria [15,16]. In context, the bioactive compounds of medicinal plants have been used in the treatment of various cancers for several centuries, which have proven the presence of various antitumor compounds in these plants [17]. Despite extensive research and reports on pomegranate in recent years, no literature review has documented studies related to the phytochemical characterization of the Bidah pomegranate cultivar or its effects as antibacterial and anticancer agents. Hence, the research aimed to identify and quantify the phytochemical compounds of the Bidah pomegranate cultivar and to evaluate its biological activity against pathogenic bacteria and the colorectal cancer cell line (Caco-2). This study highlighted the phytochemical and antioxidant capacity of the shoot and callus of Bidah pomegranate cultivar and investigated its biological activity as an antibacterial and anticancer agent. In conclusion, the findings of this study underscore the potential of Bidah pomegranate cultivar as a natural source of phytochemical compounds, antioxidants, antibacterial, and anticancer agents. Additionally, the study highlighted the importance of conserving and promoting indigenous plant cultivars and obtaining mass-propagation materials for pharmaceutical studies through the micropropagation technique. These efforts can contribute to biodiversity and sustainable agriculture in the Kingdom of Saudi Arabia. Furthermore, the research community can leverage this knowledge to further explore the therapeutic applications of Bidah pomegranate and develop novel interventions for various health conditions.

## 2. Materials and Methods

### 2.1. Chemicals

Methanol HPLC grade standards such as gallic acid, tannic acid, quercetin, glucose and plant growth regulators (PGRs), agar, and nutrient medium were obtained from Sigma-Aldrich, St. Louis, MO, USA.

### 2.2. Plant Material Collection

The mother tree of Bidah pomegranate cultivar was collected by a plant tissue expert from the Bidah area, Al-Bahah region of Saudi Arabia, in 2022, by following the international standards, guidelines, and legislation [18].

### 2.3. Explants and Media Preparation

Cuttings of about 5 cm of Bidah pomegranate cultivar were taken as explants from the mother tree. These cuttings were then washed thoroughly to remove unwanted debris and disinfected using the following protocol. The explants were immersed in 25% (*v*/*v*) Clorox solution for 20 min, followed by 0.1% mercuric chloride for 3 min, and then in 70% ethanol for 1 min. They were subsequently washed three times with sterile distilled water under the sterile conditions of a Class-II-B2-biological-safety-cabinet (Biobase, Jinan, China). The cuttings were inoculated into Murashige and Skoog medium (MS), Woody Plant Medium (WPM), and CHU (N6) Medium. These media were supplemented with different concentrations of 6-Benzylaminopurine (BAP) and 2,4-dichlorophenoxyacetic acid (2,4-D), sucrose as a carbon source (30 g/L), and 7 g/L of agar, and the pH was adjusted to 5.7 before autoclaving at 121 °C for 20 min. The plant cultures were incubated for three months under optimal growth conditions (25 °C ± 1, with 16/8 h illumination periods) for shoot and callus induction.

### 2.4. Phytochemical Analysis

#### 2.4.1. Extraction of Phytochemicals

The dried shoot and callus of the Bidah pomegranate cultivar were ground using a blender. After that, two grams of the powdered materials were placed in 100 mL of methanol HPLC grade for extraction. The extraction process was performed using an Innova 44 Inc. incubator for 2 days at 107 rpm, with the temperature set at 25 °C. Thereafter, the obtaining mixture was centrifuged at 5000 rpm for 15 min to separate the organic and aqueous phases. Subsequently, the supernatant was collected and dried at room temperature. The resulting powder was dissolved in methanol HPLC grade and filtered using a 0.45 µm nylon syringe filter for phytochemical analysis.

##### GC/MS Analysis

The phytochemical compounds of the methanolic extract of pomegranate shoot and callus were identified using gas chromatography/mass spectrometry (An Agilent 7890B, Santa Clara, CA, USA). In total, 2 µL of the extract was injected via an autosampler injection system of GC/MS. For the separation of phytochemical compounds, a DB-5 MS capillary column from Agilent technologies (30 m length × 0.25 mm internal diameter, phase thickness 0.25 μm) was used. The helium gas was used as carrier at a constant flow rate of 1 mL/min and the split flow rate was 25 mL/min. The oven temperature was set from 60 °C to 280 °C and the injector temperature was kept at 250 °C with a total run time of 61 min. The transfer line temperature was adjusted at 250 °C. The MS detector was set as follows: Acquisition scan type, mass ranging from 50 to 1000 g/mol, scan speed 1.56, 4-min solvent delay, and 230 °C MS source temperature. The identification of phytochemicals in the shoot and callus extract of Bidah pomegranate cultivar was achieved using the retention time and commercial libraries of the National Institute of Standards and Technology (WILEY 9th edition, NIST-08 MS library, Gaithersburg, MD, USA).

##### Total Phenol Content Determination

The total phenolic content (TPC) in the samples of shoot and callus of pomegranate was estimated using the Ainsworth and Gillespie [19] method with slight modifications. Overall, 100 μL of the extracted pomegranate materials was mixed with an equal volume of Folin–Ciocalteu reagent and neutralized by 300 µL of Na_2_CO_3_ solution (20%) (*v*/*v*). Thereafter, the obtained combination was incubated for 20 min in the dark at 25 °C. Methanol was used as a blank solvent on the UV–visible spectrophotometer in both the reference standard and the extracted sample of pomegranate. The optical density was measured at 765 nm using a UV–visible spectrophotometer (UV-1800, SHIMADZU, Kyoto, Japan). For the TPC determination, the equation according to the standard curve of gallic acid, which is prepared by using different concentrations of the standard (gallic acid), was used. The TPC was calculated as gallic acid equivalent per gram of sample dry weight (GAE)/g of DW.

##### Total Flavonoid Content Determination

The total flavonoid content (TFC) in the shoot and callus extract of Bidah pomegranate cultivar was estimated using the method by Ordoñez et al. [20], with minor changes as follows: a volume of 0.5 mL of pomegranate Ordoñez methanolic extract was mixed well with 0.5 mL of 2% aluminum chloride. Then, the mixture was kept in the dark for 20 min at 25 °C. Methanol was used as a blank solvent, while the absorbance of the pomegranate materials extracted was measured spectrophotometrically at 420 nm. The calibration curve was built using several concentrations of quercetin. The obtained equation depends on the regression curve used for the TFC determination, which is defined as a quercetin equivalent per gram of sample dry weight (QE)/g of DW.

##### Total Tannin Content Determination

The total tannin content (TTC) in the pomegranate materials extract was determined using the Folin–Ciocalteu method [21] with minor modifications as described before [22]. In total, 0.1 mL of the pomegranate materials extract was added to a tube containing 0.1 mL of Folin–Ciocalteu phenol reagent and 1.5 mL of deionized water for eight min. Then, a 0.3 mL of sodium carbonate solution (35%) was added. The solution obtained was shaken and incubated at 25 °C in a dark condition for 20 min. The resulting color was measured at 700 nm. The linear equation depends on the regression curve constructed by using the tannic acid standard used for TTC estimation. The generated amount of tannic acid is defined as tannic acid equivalent per gram of sample dry weight (TAE)/g DW.

### 2.5. Total Carbohydrate Content Estimation

The total carbohydrate content in the shoot and callus extract of Bidah pomegranate was determined by adopting the following method [23]: 1 mL of phenol (5%) and 5 mL of sulfuric acid (96%) were added to 1 mL of pomegranate extract and incubated in a water bath set at 30 °C for 20 min. The absorbance was recorded at 490 nm using a UV–visible spectrophotometer. The regression calibration was bolting from glucose, and the linear equation was used for the total carbohydrate determination, which is defined as glucose mg/g DW.

### 2.6. Antioxidant Capacity

#### 2.6.1. Diphenyl-2-picryl-hydrazyl (DPPH) Assay

The DPPH scavenging activity of pomegranate extract was determined using the DPPH assay method [24]. A solution of 0.1 mmol L^−1^ DPPH in methanol was prepared. Then, about 750 µL of the pomegranate extract was added to 750 µL of the DPPH solution. The mixture was incubated for 20 min at 25 °C and the absorbance was measured at 517 nm. The percentage of the DPPH scavenging activity of the extract was estimated using the following formula: DPPH scavenging activity (%) = [(absorbance of control − absorbance of sample extract)/absorbance control] × 100, where absorbance of control is the absorbance of methanol+ DPPH and absorbance of sample is the absorbance of DPPH radical + sample extract.

#### 2.6.2. Total Antioxidant Activity (TAC) Determination

The total antioxidant activity of pomegranate shoot and callus extract was estimated using the Prieto et al. [25] described method. A volume of 0.15 mL of the pomegranate sample (4 mg/mL) was mixed with 1.5 mL of the reagent solution of 0.6 M sulfuric acid, 28 mM sodium phosphate, and 4 mM ammonium molybdate. The reaction mixture was incubated at 95 °C for in a water bath for 90 min. The optical density of the mixtures was measured at 695 nm. The TAC was expressed as milligrams of ascorbic acid equivalents per gram of pomegranate extract.

#### 2.6.3. Ferric Reducing Ability of the Plasma (FRAP) Assay

The iron-reducing power of pomegranate extract was assessed following the method outlined by Zhao et al. [26]. In this procedure, 500 µL of the extract (4 mg/mL) was combined with 2.5 mL of 200 mmol/L phosphate buffer (pH 6.6) and 2.5 mL of 1% potassium ferricyanide. The mixture was then incubated at 50 °C for 20 min. After incubation, 2.5 mL of 10% trichloroacetic acid was added, and the tubes were centrifuged at 10,000 rpm for 10 min. Subsequently, 5 mL of the upper layer was mixed with 5 mL of distilled water and 1 mL of 0.1% ferric chloride. The absorbance of the resulting reaction mixtures was measured at 700 nm. The ferric reducing power assay was expressed as milligrams of ascorbic acid equivalents per gram of extract.

### 2.7. Microbial Susceptibility Testing of Bidah Pomegranate Extract

The *Staphylococcus aureus* (ATCC 2913) and *Escherichia coli* (ATCC 36218), which were used in this experiment, were obtained from the Microbiology Department, Applied Science College, King Saud University, Riyadh, Saudi Arabia. The investigated bacteria were inoculated in nutrient broth media overnight at 37 °C and adjusted to 0.5 McFarland turbidity standards. Then, the well-plate agar diffusion method was carried out by inoculating bacteria onto Sabroud Dextrose Agar (SDA) plates using a swab. A sterile cork borer was used to form wells (6 mm in diameter) on the agar plates. We then added 10 mg of plant extracts dissolved in 1 mL of deionized water to obtain a concentration of 10 mg/mL. Next, we added 0.5 mL of each plant extract and Ceftazidime (CAZ) 30 µg disc to each well in the inoculated culture plates and incubated them at 37 °C overnight [27]. The microbial susceptibility was determined by measuring the zone of inhibition (ZOI) twice for three replicates.

### 2.8. Anti-Proliferative Assay

Caco-2 cancer cell line was grown and passaged at around 95% confluence in a 10% FBS DMEM obtained from SIGMA. Overall, 50,000 cell/mL was prepared and seeded in a 96-well plate for the viability test. The crude extract was prepared as 10 mg/mL stock solution and a serial dilution for concentration levels of 1.1, 0.37, 0.12, 0.04, 0.01, 0.005, 0.002, and 0.0005 mg/mL was made and added to the cell accordingly. The crude extract incubation was kept in a 5% CO_2_ incubator at 37 °C for 72 h. before 20 µL of MTT salt (5 mg/mL) was added and incubated at 37 °C for an additional 2 h. Thereafter, the formazan crystal formed was later dissolved in isopropanol before the plate was read for optical density at 597 nm. Origin 6.1 software was used for the analysis and IC_50_ calculation.

In addition, the phenotypic effect of the crude extract was investigated. Caco-2 cells were cultured in a 6-well plate, and treatment with the crude extract at 100 µg/mL was performed at around 70% confluence. The time-point effect of the crude extract was observed using Motic AE31E inverted microscope at 12 h and 24 h post treatment. Meanwhile, an equal amount of DMSO was used in treating Caco-2 cells as a vehicular control.

### 2.9. Data Analysis

The data generated from the methanolic extract of Bidah pomegranate was subjected to statistical analysis using SPSS (version 20). One-way analysis of variance (ANOVA) was performed to separate the means and significance level determination at (*p* < 0.05). ^a,b,c^ mean within the same column differs significantly (*p* < 0.05).

## 3. Results

### 3.1. Phytochemicals of Bidah Pomegranate

**Figure 1:**
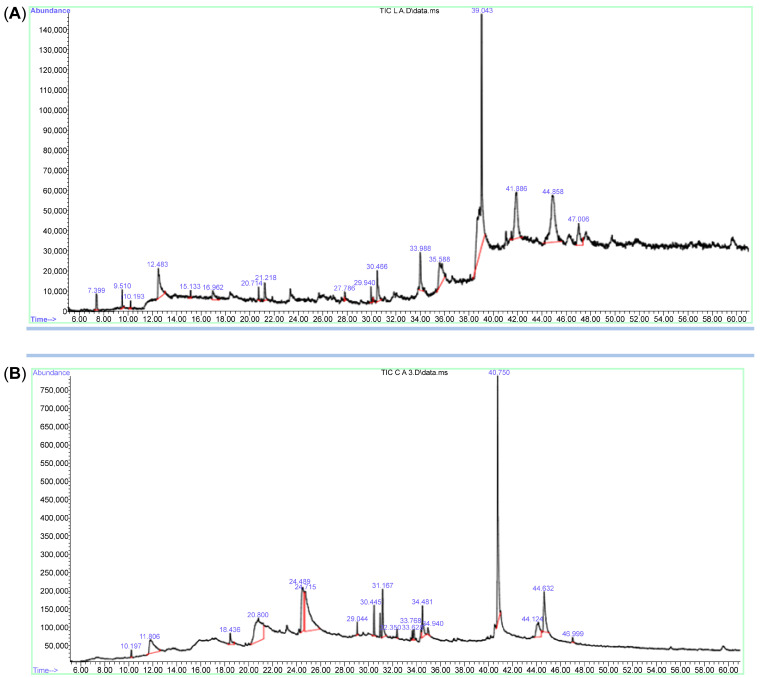
GC/MS Chromatogram of Bidah pomegranate extract (**A**) shoot extract and (**B**) callus extract.

**Figure 2:**
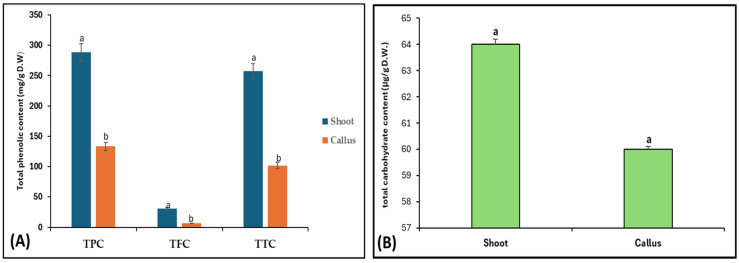
(**A**) Phenolic content (TPC, TFC, and TTC) and (**B**) total carbohydrate content of shoot and callus of Bidah pomegranate. The presented data are the mean ± standard deviation (SD). ^a,b^ the means within the same column, distinguished by different superscripts, exhibit significant differences (*p* < 0.05).

For phytochemicals identification and quantification of Bidah pomegranate cultivar, the shoot and callus methanolic extract were subjected to gas chromatography/mass spectrometry and UV–visible spectroscopy analysis. The identification of plant secondary metabolites of pomegranate materials was performed using commercial libraries, comparison of mass spectra, retention time, and matches percentage of reference compounds according to the National Institute of Standards and Technology (NIST). The results obtained from GC/MS analysis revealed about 17 photochemical compounds in shoot extract and 18 compounds in the callus extract of pomegranate linked to plant secondary metabolites (Figure 1 and Table 1). Likewise, a wide range of plant secondary metabolites such as monoterpenoid, triterpenoid, and fatty acids were recorded in Bidah pomegranate cultivar (Table 2). The estimation of phenolic components such as total phenol content (TPC), total flavonoid content (TFC), and total tannin content (TTC) in the Bidah pomegranate cultivar showed that the pomegranate contained high amounts of these compounds in both shoot and callus; whereas, the shoot extract recorded significant results of TPC (288 mg/g D.W), TFC (30.9 mg/g D.W), and TTC (257 mg/g D.W) compared to the callus extract, which showed about 133 mg/g D.W TPC, 6.6 mg/g D.W TFC, and 102 mg/g D.W (Figure 2A) and there no significant result was observed between shoot and callus in the total carbohydrate content (Figure 2B).

### 3.2. Antioxidant Activity of Bidah Pomegranate

**Figure 3:**
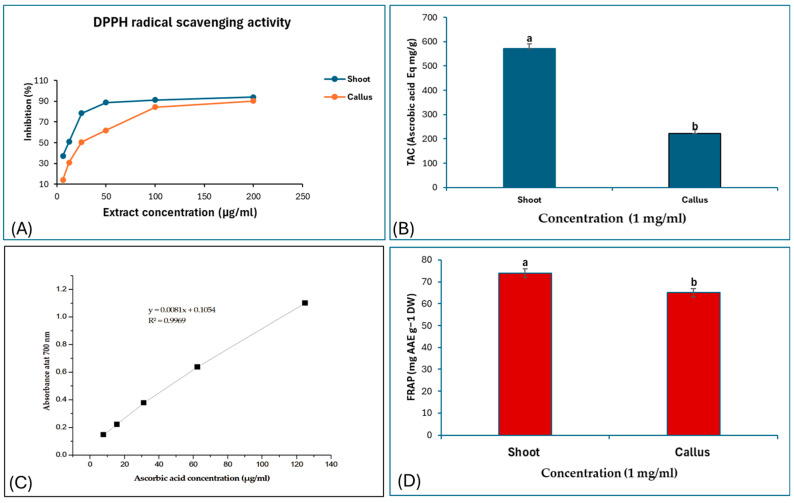
(**A**) DPPH scavenging activity (%), (**B**) total antioxidant activity Eq mg/g, (**C**) ascorbic acid standard curve, and (**D**) ferric reducing antioxidant power (FRAP) of pomegranate materials extract. The presented data are the mean ± standard deviation. ^a,b^ the means within the same column, distinguished by different superscripts, exhibit significant differences (*p* < 0.05).

Here, in this study, the antioxidant capacity of Bidah pomegranate materials extract was measured using Diphenyl-2-picryl-hydrazyl (DPPH) and the total antioxidant capacity (TAC). The DPPH results (Figure 3A) indicate that the pomegranate shoot and callus extract exhibit high antioxidant activity with a lower Half-maximal inhibitory concentration (IC50) value, the shoot extract recorded 17 µg/mL, callus extract of 19.5 µg/mL, and ascorbic acid recorded of 12 µg/mL. The highest concentration of shoot extract scavenged about 90% of DPPH free radicals, while the same concentration of the callus extract was scavenging about 80% of the DPPH free radicals (Figure 3A). The shoot extract recorded significant antioxidant capacity compared to the callus extract (Figure 3B). Also, the ferric reducing antioxidant power of Bidah pomegranate shoot and callus extract was estimated using ascorbic acid for the calibration curve (Figure 3C) and a similar result was also recorded (Figure 3D). The antioxidant capacity of the extract is attributed to the existence of high amounts of total phenolic content, total flavonoids content, and total tannin content shown in Figure 2.

### 3.3. Antimicrobial and Anticancer Assay

**Figure 4:**
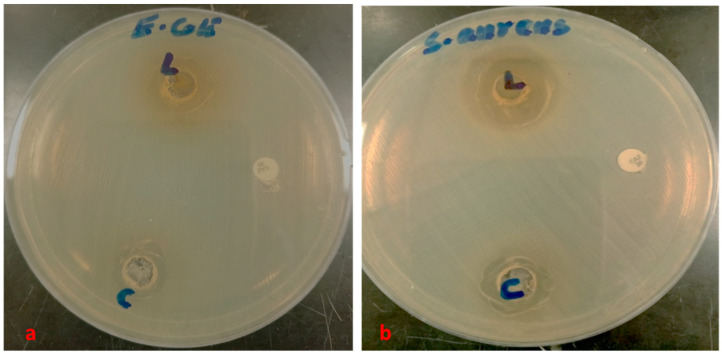
Inhibition zone diameter of Bidah pomegranate methanolic extract and the control against pathogenic bacteria (**a**) *E. coli* and (**b**) *S. aureus*, (L = Shoot extract, C = callus extract of Bidah pomegranate cultivar and Control = Ceftazidime 30 µg).

**Figure 5:**
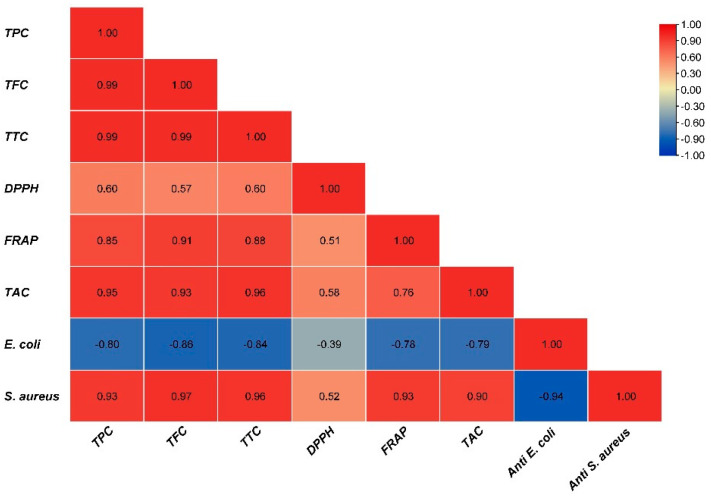
Correlation matrix heatmap of bioactive compounds, antioxidant capacity, and antibacterial activity.

**Figure 6:**
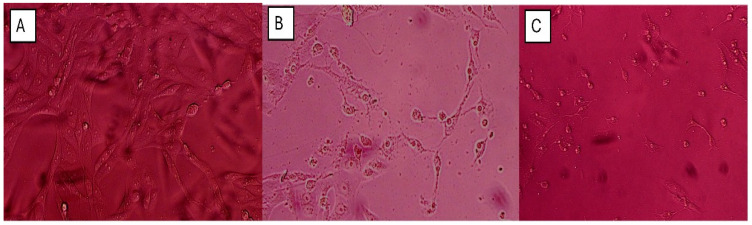
Photomicrograph showing the antiproliferative effect of Bidah pomegranate shoot extract on colorectal cancer cell line (CaCo-2): (**A**) Vehicular Control showing viable Caco-2 cells with an intact adhesion and normal morphology, (**B**) 12 h. Post treatment, showing a decreased cell population and shrink cells with reduced cytoplasm, nuclei, and loss of cell adhesion, and (**C**) 24 h. post treatment, revealing pronounced morphological changes. The morphological effect observed was performed at a 3× IC50 (≈100 µg/mL) value from the MTT assay.

**Figure 7:**
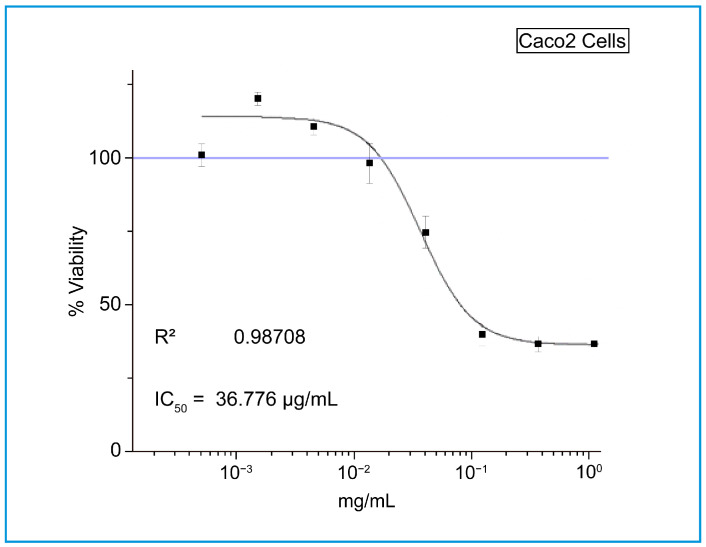
The antiproliferative effect of Bidah pomegranate shoot extract on the viability of the colorectal cancer cell line (CaCo-2).

The antibacterial effect of shoot and callus extracts of Bidah pomegranate cultivar was evaluated against pathogenic bacteria (*Escherichia coli* and *Staphylococcus aureus*). The diameter of the bacterial growth inhibition zone was measured and expressed in millimeters at the end of the incubation period. Both shoot and callus extracts of Bidah pomegranate cultivar were effective against the bacteria, as shown in Table 3 and Figure 4*. Staphylococcus aureus* is the most susceptible organism to pomegranate shoot and callus extracts, showing a 20 mm diameter inhibition zone with the shoot extract of pomegranate, while a 15 mm diameter inhibition zone was observed with the callus extract of pomegranate (Table 3 and Figure 4). Whereas, *E. coli* showed a moderate susceptibility to both shoot and callus extracts. On the other hand, all tested organisms were resistant to the commercial antibiotic (Ceftazidime 30 µg disc) (Table 3 and Figure 4). Moreover, the relationship between phytochemical compounds and their antioxidant and antibacterial activities was investigated. In Figure 5, the numbers within each cell of the matrix represent the correlation coefficient between the corresponding variables, indicating the strength and direction of linear relationship. Red color indicates a strong positive relationship between parameters, while blue signifies a strong negative relationship. In general, a strong positive relationship was observed between phenolic content (TPC, TFC, and TTC) and antioxidant assays (DPPH, RFAP, and TCA). Additionally, a strong positive correlation was found between phenolic content (TPC, TFC, and TTC) and the inhibition zone of *S. aureus*, with correlation coefficients of 0.93, 0.97, and 0.96, respectively. Conversely, a negative correlation was noted between the phenolic content and *E. coli* (Figure 5). Based on the result of antioxidant assay and the IC50 value, shoot extract of Bidah pomegranate was examined against the colorectal cancer cell line (CaCo-2). The degree of cytotoxicity of pomegranate shoot extract toward the cell lines was estimated using an MTT assay. The result revealed that pomegranate shoot extract possessed a moderate antiproliferative property against the cancer cell line (Figure 6) as evidenced by its IC50 value of 36.776 µL/mL (Figure 7). Further exploration of the phenotypic effect at 3× IC50 suggests that the crude extract might possess some apoptosis induction properties due to the reduced/shrinking cells observed (Figure 6).

## 4. Discussion

Medicinal plants are now considered a natural source of bioactive compounds that are used as antimicrobial and anticancer agents due to their antioxidant and anti-mutagenic activities, along with their low side effects, low cost, and availability [54,55]. These compounds include phenols, alkaloids, tannins, flavonoids, terpenoids, and their derivatives [56]. Plants produce specialized secondary metabolites as part of their defense mechanism, the most prominent of which are phenolics, flavonoids, alkaloids, lignoles, saponins, cardiac glycosides, and terpenoids [57,58]. Pomegranate is one of the medicinal plants that contains a wide spectrum of bioactive constituents [59]. It has been used for generations to treat various diseases such as ulcers, male infertility and diarrhea. It also possesses anti-diabetic, antitumor, anti-inflammatory, anti-malaria, anti-fibrotic, antifungal, and antibacterial properties, among other effects [4]. Bidah pomegranate is a unique cultivar adapted to Saudi Arabia conditions and is being used in traditional medicine for treating diseases. Since there are no documented data related to its phytochemicals and biological activity, this study shed light on the importance of the Bidah pomegranate cultivar. The phytochemical identification of Bidah pomegranate cultivar revealed a wide range of plant secondary metabolites, including monoterpenoid, triterpenoid, and fatty acids, as shown in Table 2. Some of these identified secondary metabolites in the Bidah pomegranate cultivar have been previously reported in pomegranates. For instance, linolenic acid was found in the pomegranate leaf and seed oil [9,60]. Moreover, the identified phytochemicals include important bioactive constituents with strong biological activity such as antimicrobial and antitumor, according to previous reports as detailed in Table 1. While the quantification of phenolic content demonstrated that the Bidah pomegranate cultivar contains a high amount of phenolic compounds, total phenolic content (TPC), total tanin content (TTC), and total flavonoid content (TFC) (Figure 2), these findings are in alignment with previous reports about pomegranates [61,62,63]. The level of phenolic content (total phenols, flavonoids, and tannins) in the Bidah pomegranate cultivar is higher than the previously reported literature on pomegranates [64], which recorded 199 mg GAE g^−1^ DW total phenol content, 24 mg CATE g^−1^ DW flavonoids, and 99 mg ECE g^−1^ DW total tannin content of hydro-methanolic leaf extracts of *P. granatum* (pomegranate). The phenolic composition of the plant can be significantly influenced by various factors, including climatic, agronomic, and genomic conditions, as well as pre- and post-harvest stages [65]. For the antioxidant capacity of Bidah pomegranate, the DPPH assay was used to measure the scavenging activity of antioxidants in the pomegranate sample. As shown in Figure 3A, the extracts exhibited strong scavenging activity, recording about 17 µg/mL IC50 for the shoot extract and 19.5 µg/mL IC50 for the callus extract, which reflects the amount of phenolic content in the cultivar. The IC50 is the concentration of an antioxidant-containing substance required to scavenge 50% of the initial DPPH radicals. The lower the IC50 value, the more potent the substance is at scavenging DPPH, indicating higher antioxidant activity [66]. This potent antioxidant activity helps in neutralizing free radicals, thereby reducing oxidative stress and preventing chronic diseases.

Particularly, variation in secondary metabolites has been observed between the shoot and callus extract of Bidah pomegranate. The phytochemical compounds detected in the shoot extract of Bidah pomegranate differ completely from those identified in the callus extract, as shown in Table 1. The induction of callus from vegetative parts of a plant can increase the bioactive compounds or even generate new ones. For example, it has been stated that callus cultures of *Byrsonima verbascifolia* (L.) DC. are a new of source of bioactive compounds [67]. Similarly, in African pencil cedar (*Juniperus procera*), 2,4-D was effective in callus induction and resulted in different bioactive compounds compared to naturally grown plants [68]. Additionally, in a study on *Gazania rigens* (L.) Gaertn., 2,4-D was used to induce callus formation, which led to the production of bioactive compounds like costunolide [69]. In this context, it has been stated that 2,4-D induces bioactive compound production [70]. 2,4-Dichlorophenoxyacetic acid (2,4-D), which has been used to transform vegetative parts into callus form in micropropagation techniques, might upregulate some genes related to secondary metabolite synthesis as well as downregulate others. The Bidah pomegranate shoot and callus extracts demonstrated high antioxidant activity (Figure 3) and effectiveness against human pathogenic bacteria (Figure 4 and Table 2). Correspondingly, when the geometric mean values of the growth inhibition zone in two independent assays were equal to or larger than 10 mm in diameter, the antimicrobial activity was considered positive [71,72]. The antimicrobial activity of pomegranate is related to the total tannin and flavonoid content [73]. The findings of this current study are consistent with previous reports, which stated that the crude extract of pomegranate demonstrated effective antimicrobial activity, as evidenced by its inhibitory effect on the bacterial growth of two important human pathogens (*S. aureus* and *E. coli*) [2]. The antibiotic properties of pomegranate extracts are of extreme interest considering the ongoing threat of bacterial strains developing resistance to conventional antibiotics. Moreover, the Bidah pomegranate shoot extract showed antiproliferative effects against the growth of the colorectal cancer cell line and the effect raised with shoot extract concentration (Figure 6 and Figure 7). Several morphological changes have been observed in colorectal cancer cells (CaCo-2) treated with pomegranate shoot extract. These alterations include nuclear fragmentation, membrane damage, cell shrinkage, and a reduction in cell size, all of which are indicative of programmed cell death. The effect of Bidah pomegranate shoot extract on colorectal cancer cells (CaCo-2) may be due to the presence of lupeol, which has strong antitumor activity [74]. Consistently, lupeol shows great potential in the prevention and treatment of colorectal cancer [49]. There is growing evidence indicating that pomegranate targets a wide range of genes and proteins to inhibit the growth and progression of cancer cells [75]. Moreover, the biological activity of phenolic compounds in cancer extends beyond their role in cell defense to also exert specific mechanisms at early cancer stages, which include cell signaling, epigenetic actions, hormonal/enzyme regulation, and immunoenhancing activity [76]. As the main objective of this section is to evaluate the Bidah pomegranate extract against cancer cell lines, no positive control was used for assessing anticancer activity, which may provide a clearer understanding of the extract’s activity. Generally, this study highlighted the phytochemicals and potential biological effects of the shoot and callus of the Bidah pomegranate cultivar. Further studies could explore its mechanism of action, effectiveness on other cancer cell lines, and potential use in combination with other treatments. Additionally, isolation and purification of specific bioactive compounds should be considered.

## 5. Conclusions

In summary, Bidah pomegranate cultivar grown in the Al-Bahah region in the kingdom of Saudi Arabia showed a wide spectrum of phytochemical compounds and antioxidant capacity, which reflect the importance of this cultivar. Based on the antibacterial activity investigation, it can be concluded that the shoot extract of Bidah pomegranate is the most effective, as indicated by its extensive zone of inhibition values against *S. aureus*, demonstrating strong antibacterial activity. Additionally, the crude extract of Bidah pomegranate shoots shows potential as an anticancer agent. Thus, Bidah pomegranate cultivar could be a promising candidate for further research as antimicrobial and anticancer agents. The current project aims to develop a micropropagation protocol for the Bidah pomegranate cultivar, contributing to sustainable agriculture. So far, only the shoot and callus have been investigated. Furthermore, the functional potential of the fruit and peel of this cultivar will be explored. Additionally, studies should investigate its mechanism of action, effectiveness on other cancer cell lines in comparison to positive control, and its potential use in combination with other treatments.

## Figures and Tables

**Table 1:** Main bioactive compounds of shoot and callus of Bidah pomegranate cultivar.

Shoot Compounds	RT (min)	MW (g/mol)	Area %	Activity	Callus Compounds	RT (min)	MW (g/mol)	Area %	Activity
4,4-Dimethyl-cyclohex-2-en-1-ol	7.399	126	1.47	N/A	9-Oxabicyclo[3.3.1]nonan-2-one	10.197	156	0.49	treating neurodegenerative [28]
4-sec-Butoxy-2-butanone	9.510	144	1.13	N/A	4H-Pyran-4-one	11.806	144	6.40	immunomodulatory, Antitumor, sarcoidosis, antioxidant, antibacterial [29,30]
1-Heptanol, 2-propyl	10.193	158	0.54	Antimicrobial [31,32]	7-Ethyl-4-decen-6-one	18.436	182	1.78	
Levomenthol	12.483	156	6.08	Antipruritic, antitussive and antispasmodic drug [33]	d-Mannose	20.800	180	15.42	immunostimulatory, anti tumor and antibacterial activity [34,35]
2-Decanone	15.133	184	0.54		L-Glucose	24.489	180	9.20	N/A
Undecanal	16.962	170	2.09	Antifungal [36]	l-Gala-l-ido-octonic lactone	24.715	238	17.58	Antibacterial.
1-Octanol	20.714	158	0.63	N/A	Estra-1,3,5(10)-trien-17β-ol	29.044	256	0.657	N/A
Propanoic acid	21.218	200	1.54		Hexadecanoic acid, methyl ester	30.445	270	1.55	Anti microbial Antioxidant and nematicides, [37,38,39]
1-Hexadecanol	27.786	242	0.69	Antioxdant [40,41]	n-Hexadecanoic acid	31.167	256	4.09	[39]
1-Dodecanol	29.940	228	0.93	N/A	d-Gala-l-ido-octonic amide	32.358	255	0.47	N/A
Tridecanoic acid	30.466	228	3.40	N/A	7-Methyl-Z-tetradecen-1-ol acetate	33.625	268	0.46	N/A
7-Hexadecenal	33.988	238	4.03	N/A	9-Octadecenoic acid,	33.768	282	1.00	Cancer preventive and antiinflammatory [42,43]
Neoisolongifolene-8-ol	35.588	220	7.24	N/A	12-Methyl-E,E-2,13-octadecadien-1-ol	34.481	280	3.68	Preventative effect against cardiovascular diseases [44]
9-Octadecenamide	39.043	281	33.18	Antimicrobial, [39]	trans-13-Octadecenoic acid	34.940	282	0.62	Anti-inflammatory, dermatitigenic, insecticides and flavour [45,46]
Lupeol	41.886	426	12.56	antioxidant,anti-topoisomerase andantitumor [39,47,48,49]	Hexadecanoic acid	40.750	330	24.44	Antimicrobial [50]
β-Sitosterol	44.858	414	19.18	Anticbacterial [51]	6,9,12,15-Docosatetraenoic acid, methyl ester	44.134	346	4.749	Antimicrobial and antibiofilm [52,53]
(2R,3R,4aR,5S,8aS)-2-Hydroxy-4a,5-dimethyl-3-(prop-1-en-2-yl)octahydronaphthalen-1(2H)-one	47.006	236	4.67	Antiinflammatory [42]	Octadecanoic acid	44.632	358	6.88	N/A
					Linolenic acid	46.999	352	0.44	N/A

**Table 2:** Molecular formula and secondary metabolites classification of shoot and callus of Bidah pomegranate cultivar.

Shoot Compounds	Molecular Formula	Secondary Metabolites	Callus Compounds	Molecular Formula	Secondary Metabolites
4,4-Dimethyl-cyclohex-2-en-1-ol	C_8_H_14_O	Alicyclic alcohol	9-Oxabicyclo[3.3.1]nonan-2-one	C_8_H_12_O_3_	bicyclic ether
4-sec-Butoxy-2-butanone	C_8_H_16_O_2_	Ketone	4H-Pyran-4-one	C_6_H_8_O_4_	Pyranone
1-Heptanol, 2-propyl	C_10_H_22_O	Branched alcohol	7-Ethyl-4-decen-6-one	C_12_H_22_O	ketone
Levomenthol	C_10_H_20_O	Monoterpenoid	d-Mannose	C_6_H_12_O_6_	Aldose
2-Decanone	C_12_H_24_O	Ketone	L-Glucose	C_6_H_12_O_6_	Aldose
Undecanal	C_11_H_22_O	Aliphatic aldehyde	l-Gala-l-ido-octonic lactone	C_8_H_14_O_8_	carbohydrate derivative
1-Octanol	C_10_H_22_O	Aliphatic alcohol	Estra-1,3,5(10)-trien-17β-ol	C_18_H_24_O	estrogen
Propanoic acid	C_12_H_24_O_2_	Carboxylic acid	Hexadecanoic acid, methyl ester	C_17_H_34_O_2_	Fatty acid
1-Hexadecanol	C_16_H_34_O	Fatty alcohol	n-Hexadecanoic acid	C_16_H_32_O_2_	saturated fatty acid
1-Dodecanol	C_15_H32O	Fatty alcohol	d-Gala-l-ido-octonic amide	C_8_H_17_NO_8_	A carbohydrate derivative
Tridecanoic acid	C_14_H_2_8O_2_	saturated fatty acid.	7-Methyl-Z-tetradecen-1-ol acetate	C_17_H_32_O_2_	lipids
7-Hexadecenal	C_16_H_30_O	Unsaturated aldehyde	9-Octadecenoic acid,	C_18_H_34_O_2_	monounsaturated fatty acid
Neoisolongifolene-8-ol	C_15_H_24_O	Cyclic alcohol	12-Methyl-E,E-2,13-octadecadien-1-ol	C_19_H_36_O	Unsaturated alcohol
9-Octadecenamide	C_18_H_35_NO	Unsaturated fatty acid amide	trans-13-Octadecenoic acid	C_18_H_34_O_2_	trans-fatty acid.
Lupeol	C_30_H_50_O	Triterpenoid	Hexadecanoic acid	C_19_H_38_O_4_	Fatty acid
β-Sitosterol	C_29_H_50_O	phytosterol	6,9,12,15-Docosatetraenoic acid, methyl ester	C_23_H_38_O2	polyunsaturated fatty acid ester
(2R,3R,4aR,5S,8aS)-2-Hydroxy-4a,5-dimethyl-3-(prop-1-en-2-yl)octahydronaphthalen-1(2H)-one	C_15_H_24_O_2_	sesquiterpenoid	Octadecanoic acid	C_21_H_42_O_4_	Saturated fatty acid
			Linolenic acid	C_21_H_36_O_4_	Fatty acid

**Table 3:** The antibacterial activity of the Bidah pomegranate methanolic extract against pathogenic bacteria showing the inhibition zone (mm).

Pomegranate		Shoot Extract	Callus Extract	Control (CAZ 30 µg)
	Bacteria			
*S. aureus*		20 ± 1.0 ^a^	15 ± 0.7 ^a^	0
*E. coli*		8 ± 0.4 ^b^	10 ± 0.5 ^b^	0

The presented data are the mean ± standard deviation. ^a,b^ the means within the same column, distinguished by different superscripts, exhibit significant differences (*p* < 0.05).

## Data Availability

The data used or analyzed in this present study are available from corresponding author.
